# Ceramide in Stem Cell Differentiation and Embryo Development: Novel Functions of a Topological Cell-Signaling Lipid and the Concept of Ceramide Compartments

**DOI:** 10.1155/2011/610306

**Published:** 2010-12-29

**Authors:** Erhard Bieberich

**Affiliations:** Program in Developmental Neurobiology, Institute of Molecular Medicine and Genetics, School of Medicine, Medical College of Georgia, 1120 15th Street Room CA4012, Augusta, GA 30912, USA

## Abstract

In the last two decades, the view on the function of ceramide as a sole metabolic precursor for other sphingolipids has completely changed. A plethora of studies has shown that ceramide is an important lipid cell-signaling factor regulating apoptosis in a variety of cell types. With the advent of new stem cell technologies and knockout mice for specific steps in ceramide biosynthesis, this view is about to change again. Recent studies suggest that ceramide is a critical cell-signaling factor for stem cell differentiation and cell polarity, two processes at the core of embryo development. This paper discusses studies on ceramide using *in vitro* differentiated stem cells, embryo cultures, and knockout mice with the goal of linking specific developmental stages to exciting and novel functions of this lipid. Particular attention is devoted to the concept of ceramide as a topological cell-signaling lipid: a lipid that forms distinct structures (membrane domains and vesicles termed “sphingosome”), which confines ceramide-induced cell signaling pathways to localized and even polarized compartments.

## 1. Ceramide: Taming of the Sphinx

When the “father of neurochemistry”, the German pathologist Johann Ludwig Wilhelm Thudichum (1829–1901), coined the term sphingolipids at the end of the 19th century, he must have thought of the Sphinx, a mythological creature testing human wit by asking riddles (which ended deadly if not answered correctly). Indeed, defining the cell-signaling function of the sphingolipid ceramide is like trying to solve a riddle posed by the Sphinx (without the grim ending, hopefully). The discovery that ceramide has a cell-signaling function goes back by more than 20 years. Interestingly, the first studies on ceramide elevation by serum deprivation or vitamin D incubation rather focused on the role of ceramide in cell cycle arrest and differentiation [1–3]. It was a few years later that ceramide emerged as one of the key factors inducing apoptosis [4–6]. 

 Since then, several thousand publications have focused on ceramide-inducing apoptosis, while much fewer studies were concerned with other functions of ceramide. This was mainly due to the limited availability of genetic approaches to alter ceramide metabolism and the rather broad group specificity of ceramide analogs and biosynthesis inhibitors. In particular, studies in Dr. Alfred Merrill's laboratory have shown that although inhibitors such as myriocin (serine palmitoyl transferase) and fumonisin B1 (ceramide synthases) are specific for their target enzymes, the inhibitory effect leads to the depletion of many ceramide species and derivatives [7–12]. Moreover, inhibiting ceramide synthase with fumonisin B1 can also result in the elevation of sphingosine (dihydrosphingosine), which itself can be toxic for various cell types. Therefore, loss-of-function experiments using these inhibitors do not accurately pinpoint which sphingolipid is instrumental in apoptosis (or other biological effects). On the other hand, gain-of-function experiments using ceramide analogs are troubled by the low water solubility of compounds whose structure comes close to that of natural ceramide (C16–C24 ceramide). In addition, water-soluble short-chain ceramides such as C6 or C8 ceramide can be recycled and converted to long chain ceramides [13–15]. Therefore, biological effects lost or regained could have been attributed to various ceramide species and derivatives. However, a particular combination of ceramide biosynthesis/metabolism inhibitors and ceramide or ceramide analogs successfully demonstrated the cell-signaling function of ceramide. When cells are preincubated with the ceramide synthase inhibitor fumonisin B1, addition of distinct ceramide species or ceramide analogs can restore the ceramide-related phenotype. Because *de novo* biosynthesis as well as the recycling pathway is inhibited, exogenously added ceramide is not converted to other ceramide species (although degradation and conversion to ceramide derivatives is still possible). Exceptionally powerful is the combination of myriocin or fumonisin B1 with ceramide analogs that are not convertible to other sphingolipids. Using this combination of reagents, our laboratory has shown for the first time that specific ceramide species are vital for the formation and differentiation of embryonic epithelia [16, 17].

## 2. Differentiating Stem Cells and the Bright Side of Ceramide

**Figure 1:**
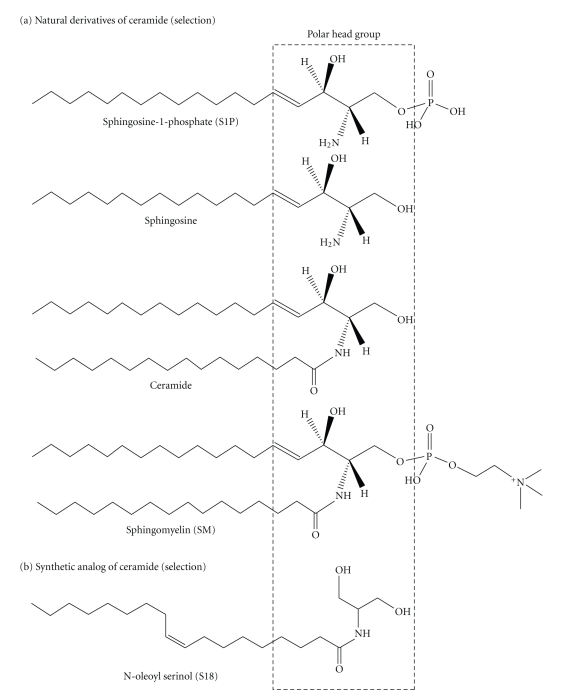
Structure of ceramide and its derivatives. (a) Natural derivatives of ceramide. All of the sphingolipids are derived from the condensation reaction of serine with palmitoyl-CoA, which is followed by reduction, acylation, and desaturation reactions to yield ceramide. In addition to the derivatives shown, glucosyl- or galactosylceramide and ceramide-1-phosphate are important structural and cell-signaling lipids, in particular for myelin formation and inflammation. (b) Synthetic analog of ceramide. The polar serine head group is preserved in ceramide and many sphingolipid analogs (only one analog (S18) is shown here). This minimal structural motif is composed of two hydroxyl groups *β*-positioned to an amino group or an imino group, which is linked to a hydrocarbon chain (dashed box, S18 or N-oleyl serinol is a derivative of 2-amino 1,3-propanediol).

 Embryonic stem (ES) cells *in vitro* differentiated to neural cell types undergo phenotypical changes that recapitulate embryo development. In the first differentiation step called germ layer formation, nonpolarized stem cells form embryoid bodies that are composed of a two-layer sphere: an outer primitive endoderm and an inner primitive ectoderm layer [16]. Since the primitive ectoderm is the primordial epithelium for all embryonic tissues, embryoid bodies are a bona fide *in vitro* model for the acquisition of germ layer cell polarity. Apicobasal cell polarity of primitive ectoderm cells is crucial for the organization and morphogenesis of the three embryonic germ layers: endoderm, mesoderm, and ectoderm. When embryoid bodies were depleted of ceramide by the incubation with myriocin or fumonisin B1, they did not form a primitive ectoderm layer. Instead, massive apoptosis of cells within the embryoid bodies was observed [16]. Supplementation of the culture medium with C16 ceramide or the novel ceramide analog N-oleoyl serinol (S18) prevented apoptosis and restored primitive ectoderm formation (Figure 1 for structures). These intriguing results were the first experimental evidence that ceramide was functionally involved in the regulation of embryonic cell polarity. 

**Figure 2:**
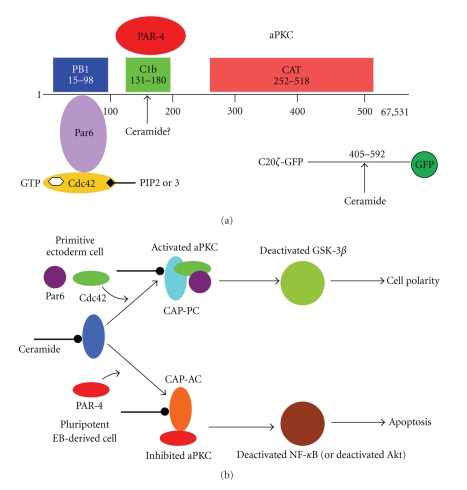
Structure of aPKC and the “flipside” model of ceramide activity. (a) In aPKC (Figure shows PKC*ζ*), the N-terminal (regulatory) and C-terminal (catalytic) moieties are connected by a hinge region. The N-terminus contains a pseudosubstrate (PS) motif and a PB1 domain. The PB1 domain is associated with the polarity protein Par6 that itself binds to Cdc42. The hinge region contains a C1b domain that has been suggested to be associated with ceramide and is a putative binding site for PAR-4. The C-terminal moiety contains the catalytic domain and several phosphorylation sites involved in activation of the enzyme. Most recently, we have constructed a dominant negative mutant from the C-terminus of aPKC (C20*ζ*-GFP) that binds to ceramide. Therefore, aPKC may contain two distinct ceramide-binding sites. In the inactive state of aPKC, the N-terminal PS motif “folds back” onto the C-terminal catalytic domain and blocks its access to protein substrates. We have proposed that binding to ceramide “opens up” aPKC and primes its activation by phosphorylation or inhibition by PAR-4 (Flipside model of ceramide activity). (b) For the activation reaction, ceramide binding to aPKC is followed by its association with Par6 and Cdc42. PIP2 or 3 may participate in aPKC activation and subcellular translocation by binding to active (GTP-associated) Cdc42 in this complex. This ceramide-aPKC polarity complex (CAP-PC) may inhibit GSK-3*β* and control cell polarity, process formation/migration, or ciliogenesis as described in Figure 3. In the presence of increased levels of active PAR-4, ceramide-associated aPKC binds to PAR-4 and forms a ceramide-aPKC apoptosis complex (CAP-AC). This complex inhibits aPKC and prevents activation of its downstream targets, NF-*κ*B and Akt. We have proposed that this leads to activation of Bax/Bad and induction of apoptosis.

 S18 is a special kind of ceramide analog designed and synthesized in our laboratory [18, 19]. By maintaining the polar (serine-derived) head group of ceramide, and at the same time, enhancing the water solubility of the lipophilic portion, we obtained a water-soluble, but still lipophilic, structural analog of ceramide (Figure 1). The extensive characterization of S18 showed that it incorporates into cell membranes and activates atypical PKC (aPKC), which was also found for ceramide [18–21]. Moreover, ceramide and S18 induce the formation of aPKC-associated complexes with polarity proteins such as Par6 and the small Rho-type GTPase Cdc42 (Figures 2(a) and 2(b)). This was shown using *in vitro* complementation assays with ceramide vesicles and purified proteins, but also in living cells visualizing S18-induced polarity protein complexes by immunocytochemistry [16, 17, 20–22]. Most excitingly, by developing a highly specific antibody against ceramide, we could show that the S18-induced protein complexes were identical to those associated with ceramide [16, 17, 23]. Therefore, our studies explained for the first time how ceramide can organize cell polarity on the molecular level. 

 Primitive ectoderm cells encompassing the lumen of the embryoid body are polarized similar to cells in the primitive ectoderm layer of the preimplantation embryo. The apical cell membrane facing the lumen or the pro-amniotic cavity is associated with F-actin and interconnected to adjacent cells via *β*-catenin. Using the anticeramide antibody, we found that in primitive ectoderm cells of embryoid bodies, the apical membrane was tremendously enriched in ceramide and codistributed with aPKC, Cdc42, F-actin, and *β*-catenin [16]. The apical membrane of the preimplantation embryo was also enriched in ceramide and codistributed with F-actin. Taken together, these results led us to design a model for ceramide-induced cell polarity. In this model, apical ceramide recruits aPKC to the membrane and organizes a cell polarity complex with Par6 and Cdc42, thereby stabilizing *β*-catenin and F-actin [22]. This was the first model combining the topological/structural properties of ceramide with a cell-signaling pathway regulating cell polarity (Figure 2(b)). 

 Cell polarity complexes associated with aPKC are not only involved in the acquisition of apicobasal polarity, but also in other cell-polarity-related processes such as cell adhesion and migration. Therefore, we tested if the function of ceramide can be extended to other aspects of aPKC-controlled cell polarity. So far, we have found that ceramide is critical for cell migration of neural progenitors, cell adhesion of epithelia, and ciliogenesis [16, 21, 22, 24]. The function of ceramide in these processes will be discussed in the following sections.

## 3. Cells on the Rest Getting Ready to Move: New Par-Tners Court for Ceramide

 From the very beginning, ceramide was found to be elevated in resting cells. As a typical response to serum deprivation/withdrawal, cells go into G0 cell cycle arrest concurrent with hydrolysis of sphingomyelin to ceramide [1, 3]. Recently, it has been shown that the elevation of ceramide in serum-deprived cells activates protein phosphatase 2a (PP2a) which then activates histone deacetylase (HDAC) and downregulates telomerase, an enzyme critical for cell cycle progression [25]. Our laboratory was interested in finding out which other properties of nonproliferating cells are regulated by ceramide. Madin Darby Canine Kidney (MDCK) cells are an exquisite *in vitro* model to study the dynamics of cell adhesion, polarity, and ciliogenesis in resting cells. MDCK cells are rapidly dividing until they reach confluence. They establish firm adhesion junctions and eventually develop a primary cilium. This cilium only appears in G0-arrested cells and disappears when cells re-enter the cell cycle. Therefore, we used confluent and serum-deprived MDCK cells to study the function of ceramide in cell adhesion and ciliogenesis. Based on our results with embryonic epithelia, we focused on the effect of ceramide on aPKC, a key factor in the assembly of polarity complexes that are critical for cell adhesion and ciliogenesis. 

 The role of ceramide binding to aPKC was determined by first generating a dominant negative mutant of aPKC (C20*ζ*-GFP), which binds ceramide but does not associate with or phosphorylate client proteins involved in cell polarity [22] (Figure 2(a)). Based on the results of binding assays using proteolytic fragments of aPKC, we narrowed down the ceramide binding site to amino acid 405–592 in the C-terminal moiety of the enzyme. This portion of aPKC does not bind to the polarity protein Par6 and does not encompass the kinase domain. Binding of ceramide to the C-terminus of aPKC was very surprising since the C1 domain (amino acid 131–180) in the regulatory portion of the enzyme was previously suggested to bind to ceramide [26, 27] (Figure 2(a)). Most recently, the C1 domain of a related protein has been found to bind to ceramide [28]. Therefore, it is likely that aPKC has two ceramide-binding sites, one in the C1 domain and another one in the C-terminus. However, based on our results, we conclude that binding to the C-terminal portion was sufficient to compete with endogenous aPKC for ceramide. We tested the effect of the dominant negative mutant using immunocytochemistry for adherens junction proteins and by monitoring transepithelial resistance [21]. Expression of the dominant-negative mutant in confluent MDCK cells compromised adherens junctions and epithelial cell contacts by affecting the intracellular distribution and protein levels of E-cadherin and *β*-catenin. The two proteins were no longer distributed to the adherens junctions, where they participate in a larger protein complex with aPKC. Since this adherens junction complex is regulated by the association of aPKC with Par6 and Cdc42, it is likely that the dominant negative mutant competed with endogenous aPKC for binding to ceramide, but it was not associated with other proteins required to form the junction complex. Consistently, depleting MDCK cells of ceramide with fumonisin B1 also impaired transepithelial resistance, which was restored by the addition of S18 or C16 ceramide [21]. Based on these results, it is reasonable to hypothesize that the ceramide-aPKC interaction is a vital part of the entry reaction in the formation of adherens junctions in epithelial cells. 

 In the ventricular neuroepithelium of mouse embryonic brain, neural progenitors divide asymmetrically with one daughter cell staying attached to the epithelium, while the other daughter cell migrates toward the cortical layer and eventually undergoes neural differentiation. Interestingly, aPKC is critical for two apparently opposite processes related to cell polarity: cell adhesion of neural progenitors in the neuroepithelium and migration of neural progenitors away from it. We tested if the ceramide-aPKC interaction was also important for these cell-polarity-related processes in the neuroepithelium. A reliable *in vitro* assay to test the effect of a compound on motility and migration is the wounding or scratch migration assay. Cells will be grown to confluence, a gap prepared by scraping off cells, and the velocity will be determined by which cells migrate into the gap. Our results showed that depleting neural progenitors of ceramide using myriocin significantly slowed down cell migration [17]. Migration was restored by supplementing the medium with S18 or C16 and C18:1 ceramide. We used C18:1 ceramide because it was one of the major ceramide species found in neural progenitors (in contrast to C18 ceramide, the monounsaturated C18:1 ceramide is a minor species in adult brain [29]). The typical short-chain ceramide analogs used for induction of apoptosis such as C2 or C8 ceramide did not induce or restore migration. This was the first evidence that distinct ceramide species are required for aPKC-related cell polarity. In conjunction with the previous results on apicobasal polarity of primitive ectoderm cells and cell adhesion of MDCK cells, one may speculate that C16 ceramide is more important for polarity and adhesion of nonmotile cells, while C18:1 ceramide is more involved in cell migration. 

 With the advent of knockout mice for specific ceramide synthases, the focus of the ceramide field has switched from the more generalized effect of ceramide depletion or supplementation to a distinct ceramide composition. In this regard, the ceramide synthase 2 (CerS2 or lass2) knockout mouse shows a remarkable phenotype. Knockout of CerS2 results in hepatocarcinomas and myelin defects eventually leading to death of mice within two years after birth [30–32]. The complete depletion of very long-chain ceramides (C22 and C24 ceramide) in liver and brain is consistent with the substrate specificity of CerS2 for the respective acyl CoA. However, the total ceramide levels are not different from those of the wild type because loss of very long-chain ceramides is compensated by the elevation of C16 and C20 ceramide. Moreover, CerS2 knockout mice also show elevation of phospholipids such as phosphatidylethanolamine with C18:1 and C18:2 fatty acids [31]. The authors of the CerS2 knockout study found that these changes increase the membrane fluidity, which was due to the compensatory changes in the lipid composition of the mouse. It should be mentioned that these mice are devoid of C24:1 ceramide, a very long-chain ceramide that is a major species in the cell membrane and known to increase membrane fluidity because of its nonsaturation. With respect to migration of neural progenitors, it is reasonable to speculate that the exquisite properties of C18:1 ceramide to enhance migration may be due to similar effects on membrane fluidity. It is noteworthy that similar effects of nonsaturated ceramide on enhancing membrane fluidity have also been shown for cold adaptation of plants, suggesting that membrane fluidity is a parameter critically regulated by ceramide in a vast variety of organisms [33]. It will be part of our future research to determine if specific changes in the ceramide composition of the cell membrane are correlated with distinct functions during neural progenitor differentiation.

## 4. Apoptosis as Polarity Disorder: When Atypical PARtners Akt up on Bad

 While the previous sections were focused on the beneficial roles of ceramide in cell adhesion and migration, cell polarity of neural progenitors may also be disturbed by proapoptotic proteins competing with polarity proteins for binding to ceramide-associated aPKC. Apoptosis of neural progenitors may thus be a polarity disorder on the cellular and molecular level. We found that prostate apoptosis response 4 (PAR-4, not related to Par6), a pro-apoptotic protein first described in prostate cancer cells, sensitizes neural progenitors to ceramide-induced apoptosis [20, 22, 34–38]. Using *in vitro* complementation assays with ceramide vesicles, coimmunoprecipitation assays, and immunocytochemistry using an antibody against ceramide developed in our laboratory, we found for the first time that ceramide induces binding of aPKC to PAR-4 [20, 23, 35, 36]. This was consistent with similar findings in other cell types showing that binding of PAR-4 inhibits aPKC [39, 40]. In conjunction with our studies on ceramide-induced association of Par6-Cdc42 with aPKC, it is reasonable to speculate that PAR-4 and Par6-Cdc42 compete for binding to ceramide-associated aPKC (Figure 2(b)). 

**Figure 3:**
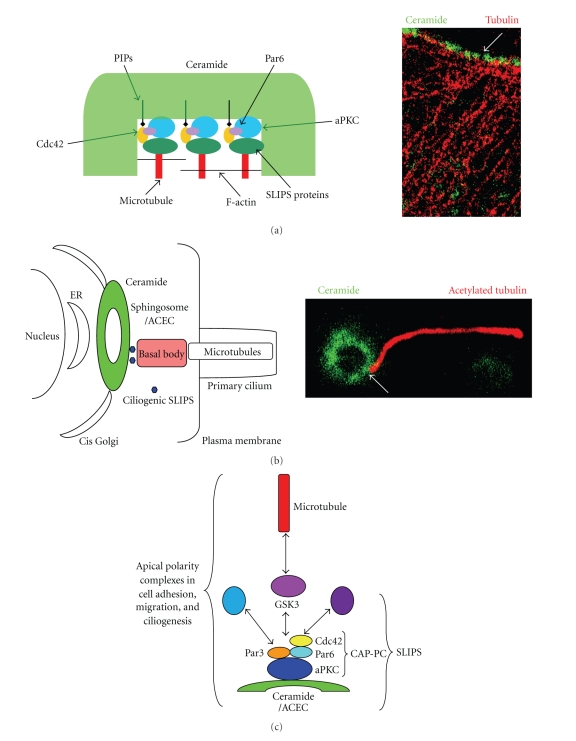
A core complex associated with ceramide may regulate cell polarity. (a) Ceramide microdomains or rafts may be formed after local hydrolysis of sphingomyelin catalyzed by acid or neutral sphingomyelinase. PIP2 or 3 embedded in these ceramide microdomains may bind to Cdc42 (via PH domain), while ceramide-associated aPKC binds to Par6. The formation of a **S**phingo**L**ipid (here ceramide)-**I**nduced **P**rotein **S**caffold (SLIPS) then regulates the dynamics of the cytoskeleton via additional proteins such as GSK-3*β*. This may sustain ceramide-rich platforms (e.g., in the apical membrane of primitive ectoderm cells) or initiate processes (“sphingopodia”) in neural stem and progenitor cells. The image on the right panel shows staining of ceramide microdomains using a ceramide-specific antibody generated in our laboratory. Note that the plus end of microtubules appears to attach to the ceramide domains. (b) Likewise, we have found that a pericentriolar, apical ceramide-enriched compartment (ACEC) appears to be attached to the basal body of the primary cilium. The right panel shows the ring-shaped structure of this compartment that we have termed “sphingosome”. (c) We hypothesize that a ceramide-aPKC polarity complex (CAP-PC) consisting of ceramide, aPKC, Par6, Cdc42, and Par3 forms a functional key element of many cell polarity-related processes including cell adhesion, process formation (“sphingopodia”), cell migration, and ciliogenesis. The local assembly of distinct sphingolipid-induced protein scaffolds (SLIPs) determines the functional specificity of this polarity complex.

 In cells with low PAR-4 expression (or inactive PAR-4), ceramide-associated aPKC nucleates a polarity protein complex by binding to Par6 and Cdc42. This will either stabilize cell adhesion (via E-cadherin and *β*-catenin) or induce migration (via inactivation of GSK-3*β*; see [17, 21] for details) (Figure 2(b)). The regulation of the balance between cell adhesion and migration involving a similar aPKC-associated protein complex is still enigmatic. It may very well be controlled by binding of aPKC to membrane lipids such as distinct ceramide or phosphatidylinositol phosphate (PIP) species, which recruit and stabilize different protein scaffolds associated with aPKC (Figure 3(a)) [22]. On the other hand, if PAR-4 expression is high (or PAR-4 is activated), the ceramide-aPKC lipid-protein complex will be inhibited by PAR-4 (Figure 2(b)). 

 In differentiating ES cells, we have found that this mechanism can eliminate unwanted tumorigenic stem cells. When embryoid bodies are plated on tissue culture dishes, the attached cells undergo neural differentiation in the absence of serum and the presence of fibroblast growth factor 2 (FGF-2). Interestingly, there is an enormous rate of cell death in the first 48 h of cultivation diminishing about half of the culture. The apoptotic cells show high expression level of Oct-4, a marker for pluripotency typical for primitive ectoderm cells, and PAR-4, the sensitizer for ceramide-inducible apoptosis [34, 35, 37]. We confirmed the critical role of PAR-4 for apoptosis by knocking-down this protein, which resulted in survival of the Oct-4-expressing cells. Moreover, supplementation of the medium with the ceramide analog S18 eliminated Oct-4/PAR-4 coexpressing stem cells [37]. This result was very exciting and significant because stem cells that retain Oct-4 expression formed tumors (teratoma) instead of differentiating and integrating into the host tissue. Elimination of these tumor stem cells by S18 is a major step in enhancing the safety of stem cell transplantation. 

 It should be noted that another group has followed up on our studies by administering liposomal ceramide to undifferentiated human ES cells [41]. This group found that ceramide can maintain the undifferentiated state by eliminating prematurely differentiated cells. On first sight, this may be contradictory to our results in that we reported the elimination of residual pluripotent stem cells by ceramide. However, residual pluripotent stem cells are not identical to undifferentiated cells because they are derived from primitive ectoderm in embryoid bodies. These cells are, similar to prematurely differentiated stem cells, dysfunctional in that they deviate from the normal path of differentiation. We have termed these cells “Zombie cells” because they remain “undead” and form tumors unless they are eliminated by the administration of ceramide analogs [37]. It should also be noted that the precise role of ceramide in stem cell differentiation is still unclear. While our studies and those from other laboratories show that ceramide eliminates improperly differentiating stem cells, recent work suggests that ceramide may also induce stem cell differentiation [35, 41–43]. As a cautionary note, however, one should distinguish between the effect of endogenous and exogenously added ceramide, in particular when using short-chain ceramide such as C2 ceramide. A comprehensive analysis of endogenous ceramide species during stem cell differentiation has shown that consistent with vast changes in the expression of different ceramide synthase isoforms, the proportion of C18, C24, and C24:1 ceramide increases while that of C16 ceramide decreases when undifferentiated ES cells mature to embryoid bodies [44]. 

 Despite this translational outcome of our work showing a method to eliminate tumor stem cells via PAR-4 as a sensitizer to ceramide analogs, the physiological function of PAR-4 for neural development still remains unclear. At first, we were intrigued by the observation that PAR-4 is asymmetrically distributed during cell division of neural progenitors. We found that the PAR-4 inheriting (or expressing) daughter cell died, while the PAR-4 (-), nestin expressing daughter cell, survived and underwent further neuronal differentiation [35]. We thought that this would be a great way to eliminate half of the neural progenitor population as observed during mouse brain development. However, since the PAR-4 knockout mouse did not show a gross embryonic phenotype, we focused on PAR-4 as a protein that induces apoptosis if inappropriately expressed during embryonic or postnatal development. We found that the pathological expression of PAR-4 facilitates ceramide-induced apoptosis in two cell types: neural crest-derived cells and astrocytes. 

 Neural crest-derived cells are the precursors for many neural and nonneural cell types. They originate from the roof plate of the closing neural tube (neural crest) and proliferate and migrate extensively throughout the embryo. Among the tissues differentiated from neural crest-derived cells are the facial bones and many supportive cells (e.g., pericytes) of the central nervous system. This shared provenance of specific skeletal and brain tissues caught our attention when we were studying the effect of ethanol on apoptosis of neural progenitor cells. In fetal alcohol syndrome, a birth defect caused by maternal consumption of alcoholic beverages during pregnancy, facial malformation, and cognitive retardation are the most obvious and severe symptoms of the newborn. Therefore, we hypothesized that neural crest-derived progenitor cells are very sensitive to ethanol, which leads to malformation of neural crest-derived tissues in fetal alcohol syndrome. Our studies showed for the first time that ethanol elevates the expression of PAR-4 and ceramide in neural crest-derived tissue culture and embryos *in vivo* [45]. Elevation of PAR-4 leading to enhanced sensitivity toward ceramide was also found in primary cultured astrocytes generated from newborn pups that carry a mutation of presenilin 1 (Psen 1), a gene mutated in familial Alzheimer's disease [46]. Taken together, these results strongly suggest that the simultaneous elevation of PAR-4 and ceramide is involved in several pathological phenotypes showing tissue degeneration due to enhanced apoptosis. 

 About ten years ago, we began to investigate which cell-signaling pathway is downstream of the ceramide-aPKC apoptosis complex (CAP-AC), and what distinguishes the cell-signaling effect of this proapoptotic lipid-protein complex from that of the ceramide-aPKC-polarity complex (CAP-PC). Based on experimental evidence obtained with neuroblastoma cells and ES cell-derived neural progenitors, our very first model suggested that CAP-AC will reduce activation of Akt/PKB and MAPK/ERK2, which will lead to activation of Bad and reduced protein levels of Bcl-2 [36] (Figure 2(b)). Bad and Bcl-2 are two BH3-only proteins counterregulating apoptosis by either facilitating or preventing the release of cytochrome c from mitochondria. In follow-up studies, we found that CAP-AC also downregulates the transcription factor NF-*κ*B, which is critical for neural progenitor cell survival [20] (Figure 2(b)). On the other hand, CAP-PC inactivated GSK-3*β*, a different cell-signaling pathway regulating the function of *β*-catenin [21] (Figures 2(b) and 3). Most recently, we published a model showing that the CAP-AC- and PC-induced cell-signaling pathways are at the cross roads of cell-signaling pathways for apoptosis and cell polarity [22]. 

 Several studies have shown that aPKC phosphorylates Akt as well as GSK-3*β*, which coregulates cell survival and polarity [47–49]. In cells with a low level of PAR-4 expression or activity; this pathway will be induced by ceramide. In cells with high level of PAR-4 expression or activity, however, this pathway is inhibited, eventually leading to apoptosis. Therefore, we hypothesize that apoptosis can be understood as a disorder of cell polarity, which may eliminate cells that do not polarize or else they would form dysfunctional or cancerous tissues. 

 It should be noted that other groups have also investigated the effect of ceramide on aPKC and Akt [50–56]. Consistent with our results, one group found that the ceramide-aPKC interaction leads to inhibition of Akt [54, 57]. However, this group has attributed the inhibitory effect to ceramide-induced activation of aPKC. It is possible that the effect of ceramide on Akt may be modulated by aPKC-interacting proteins such as Par6 and PAR-4. Recent evidence in literature suggests that an aPKC-Par6 complex activates Akt, while an aPKC-PAR-4 complex inactivates this kinase [47, 58]. Whether aPKC activates or inactivates Akt may thus critically depend on where and with which other proteins ceramide-associated aPKC interacts. This compartmentalization of ceramide and aPKC will be discussed in the following and in the last section of this paper.

## 5. Is It Time to Define a Specific Ceramide Compartment, the “Sphingosome”?

 The previous sections were concerned with the function of ceramide for individual aspects of cell polarity such as cell adhesion and migration. Most recently, we have found evidence that ceramide is involved in another phenomenon of polarized cells: ciliogenesis [24]. Non-dividing mammalian cells, regardless of residing within an epithelium or migrating, form a primary cilium. This cilium is studded with growth factor receptors, and it is assumed to act like an antenna or global positioning system, scanning the environment for sources of growth factors. When using our antibody against ceramide for immunocytochemistry with ciliated MDCK cells, we found that at the basis of the primary cilium, ceramide is highly enriched in a Golgi-derived compartment [24] (Figure 3(b)). Depletion of ceramide after establishment of cell adhesion and apicobasal polarity prevented ciliogenesis and obliterated the primary cilium. Addition of C16 ceramide or S18 to the medium restored or even enhanced ciliogenesis. Our study was the first evidence that ceramide is critical for formation or maintenance of the primary cilium. 

 It has been shown in previous studies that aPKC is critical for ciliogenesis [59, 60]. Therefore, we hypothesized that the ceramide-aPKC interaction is functionally involved in this process. Using immunocytochemistry, we found that the ceramide-enriched compartment at the basis of the primary cilium engulfed the centriole forming the basal body [24]. We also showed that the ceramide-enriched compartment was codistributed with the Golgi matrix protein GM130, a marker for cis Golgi. Most importantly, the ceramide-enriched compartment was codistributed with aPKC and Cdc42, indicating that that the core polarity complex consisting of aPKC-Par6-Cdc42 is associated with ceramide at the base of the cilium. At this point, we do not know if ceramide induces, recruits, or sustains this complex. We also do not know if ceramide is critical for the initial formation or other processes such as elongation of the primary cilium. However, it is clear from our studies that the compartmentalization of ceramide and therefore, localized formation of ceramide-associated protein complexes is instrumental in the regulation of cell polarity and ciliogenesis (Figure 3). 

 It should be noted that other groups have also found evidence for the ceramide-dependent compartmentalization of intracellular proteins. Yusuf Hannun's group has described a juxtanuclear recycling compartment termed “pericentrion” [61]. Formation of this compartment is induced by activation of PKC*α*/*β*2 with phorbol ester, and it is abolished by ceramide depletion with fumonisin B1. Although Hannun's group did not show ceramide enrichment in this compartment and found a different mechanism for the activity of ceramide (activation of PP2a leading to PKC*α*/*β*2-induced pericentrion sequestration), there are striking similarities to the apical ceramide-enriched compartment (ACEC) found in our group. It is also noteworthy that other sphingolipids have been implicated in the compartmentalization of polarized cells. Pioneering work in Kai Simmon's and Gerrit van Meer's groups demonstrated that glycosphingolipids and sphingomyelin are asymmetrically transported and distributed in polarized MDCK cells [62]. Of interest are also recent studies showing that the gangliosides GM3 and GM1 are differentially distributed to microvilli and primary cilia in polarized cells [63]. Taken together, there is growing evidence that sphingolipids, and in particular ceramide, may organize a specialized apical compartment in polarized cells. We suggest the term “sphingosome” for this compartment to include ceramide and other sphingolipids in its composition and generation (Figures 3(b) and 3(c)). We will now investigate how the sphingosome may be regulated by the distinct composition of sphingolipids and how it participates in the regulation of cell polarity and differentiation. 

## Abbreviations

ACEC: Apical ceramide-enriched compartment
aPKC: Atypical PKC
CAP-AC: Ceramide-aPKC apoptosis complex
CAP-PC: Ceramide-aPKC polarity complex
MDCK: Madin-Darby Canine Kidney
PAR-4: Prostate apoptosis response 4
SLIPS: Sphingolipid-induced protein scaffold
S18: N-oleoyl serinol.
